# Pacemaker lead dislocation during TricValve procedure with an extremely small superior vena cava

**DOI:** 10.1007/s10554-024-03067-1

**Published:** 2024-02-19

**Authors:** Mi Chen, Aris Moschovitis, Maurizio Taramasso

**Affiliations:** 1 Department of Cardiology, University Hospital of Zurich, University of Zurich, Zurich, 8091 Switzerland; 2 Heart Center Hirslanden Zurich, Witelli-kerstrasse 36, Zurich, CH-8008 Switzerland

A 49-year-old inoperable male presented for treatment due to severe tricuspid regurgitation. The heart team opted to implant a TricValve (Unimedtec) considering the challenging: impacted pacemaker lead, esophageal stenosis, and a small superior vena cava (Fig. 1, Supplementary Fig. 1).

During the procedure, a 27-Fr sheath was inserted into the right femoral vein, and a 21-mm SVC valve delivery system was introduced (Panels A). However, as the system advanced, its nose cone unintentionally pushed two pacemaker leads into the right brachiocephalic vein (RBV), causing a blockage at the confluence of the brachiocephalic veins. This resulted in the pacemaker leads being carried alongside the delivery system, creating a compounded obstruction (Panel B). To solve this, a goose snare catheter was positioned to grasp and pull the pacemaker leads inferiorly (Panel C), preventing their re-entry into the RBV. However, while the SVC delivery system was trying to reintroduce to RBV, causing increased upward tension on the ventricular electrode (Panel D), the snare system simultaneously exerted more force to pull both electrodes downward, resulting in the dislocation of the atrial electrode (Panel E). After successfully deploying the SVC valve (Panel F), the team repositioned the dislocated atrial electrode (Panels G and H) and implanted a 31-mm IVC valve. Angiography confirmed correct valve placement and function (Panel I). A new atrial electrode was then placed from left brachiocephalic vein, through the cell of the SVC valve and secured in the right atrial wall (Supplementary Fig. 2).

Post-procedure, the patient had functioning SVC and IVC valves, no pacemaker issues, and was discharged in four days (Video 1).

**Fig. 1:**
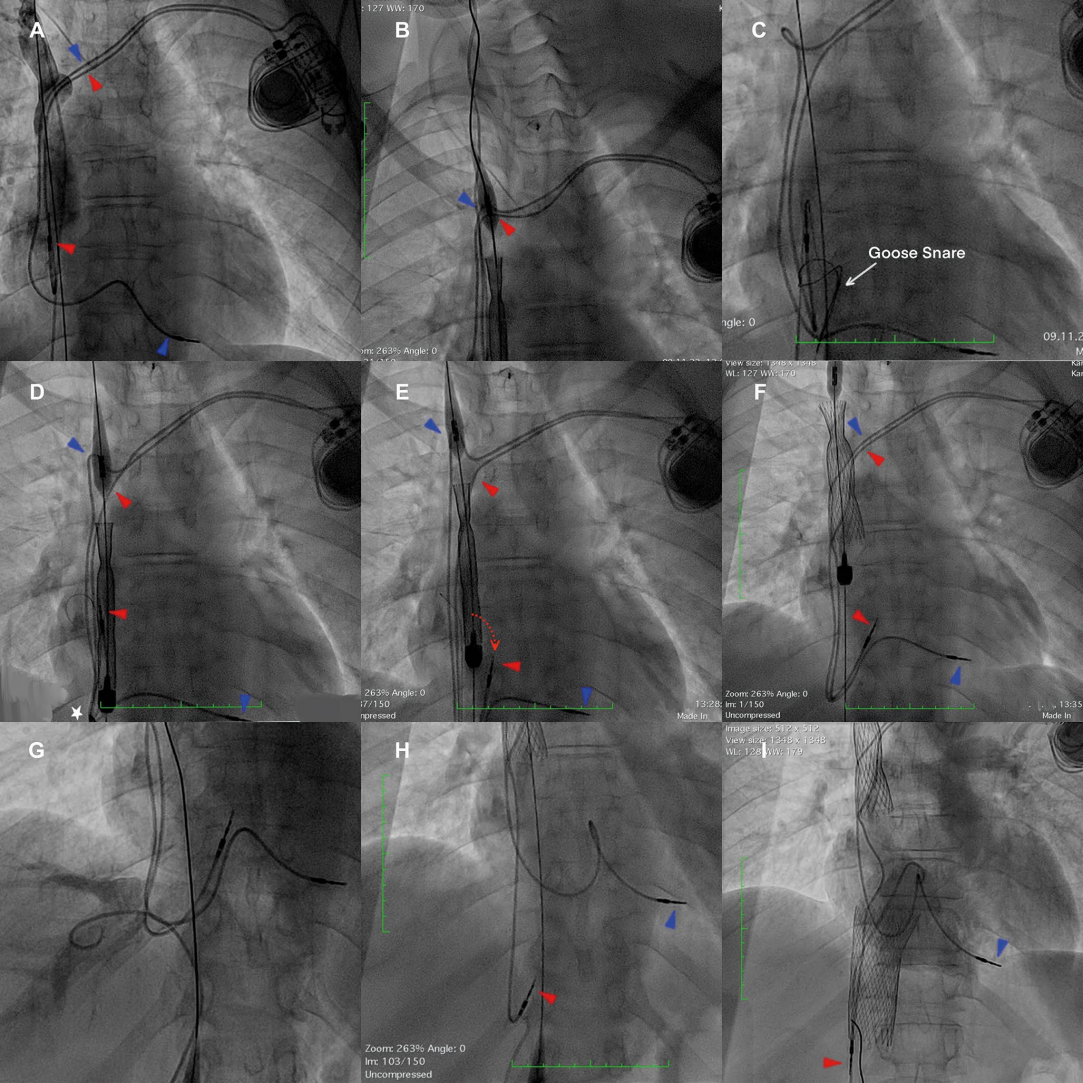
Pacemaker lead dislocation during the TricValve procedure

### Electronic supplementary material

Below is the link to the electronic supplementary material.

TricValve with pacemaker leads dislocation

## Data Availability

Data can be required from corresponding author if needed.

